# Two solutions for efficient light-harvesting in phototrophic *Gemmatimonadota*

**DOI:** 10.1128/msystems.01094-25

**Published:** 2025-12-04

**Authors:** Alastair T. Gardiner, Yibo Jin, David Bína, Maarten Joosten, David Kaftan, Izabela Mujakić, Zdenko Gardian, Pablo Castro-Hartmann, Pu Qian, Michal Koblížek

**Affiliations:** 1 Institute of Microbiology of the Czech Academy of Sciences 86863, Třeboň, Czech Republic; 2 Materials and Structure Analysis, Thermofisher Scientific, Eindhoven, the Netherlands; 3 Faculty of Science, University of South Bohemia 204738 , České Budějovice, Czech Republic; 4 Institute of Plant Molecular Biology, Biology Centre of the Czech Academy of Science 98736 , České Budějovice, Czech Republic; 5 Department of Bionanoscience, Kavli Institute of Nanoscience, Delft University of Technology 2860, Delft, the Netherlands; 6 Institute of Parasitology, Biology Centre of the Czech Academy of Science 90801, České Budějovice, Czech Republic; 7 School of Biosciences, University of Sheffield 152813, Sheffield, United Kingdom; University of Rhode Island, Narragansett, Rhode Island, USA

**Keywords:** microbiology, phototrophy, light-harvesting, energy transfer, structural biology

## Abstract

Phototrophic Gemmatimonadota represent a unique group of phototrophic bacteria that acquired a complete set of photosynthetic genes via horizontal gene transfer and later evolved independently. *Gemmatimonas* (*Gem.*) *phototrophica* contains photosynthetic complexes with two concentric light-harvesting antenna rings that absorb at 816 and 868 nm, allowing it to better exploit the light conditions found deeper in the water column. The closely related species *Gem. groenlandica*, with highly similar photosynthetic genes, harvests infrared light using a single 860 nm absorption band. The cryo-electron microscopy structure of the *Gem. groenlandica* photosynthetic complex reveals that the outer antenna lacks monomeric bacteriochlorophylls, resulting in a smaller optical antenna cross-section. The *Gem. groenlandica* spectrum is red-shifted relative to *Gem. phototrophica* due to the formation of a H-bond enabled by a different rotamer conformation of αTrp^31^ in the outer ring. This H-bond forms with a neighboring bacteriochlorophyll and increases the intra-dimer exciton coupling, affecting the exciton localization probability within the rings and increasing exciton cooperativity between the complexes. The functional consequences of the spectral shift, caused solely by a subtle conformational change of a single residue, represent a novel mechanism in which phototrophic organisms adjust their antennae for particular light conditions and enable *Gem. groenlandica* to grow higher in the water column where more photons are available.

**IMPORTANCE:**

The photoheterotrophic species of the phylum Gemmatimonadota employ unique photosynthetic complexes with two concentric antenna rings around a central reaction center. In contrast to other phototrophic species, these organisms have not evolved any regulatory systems to control the expression of their photosynthetic apparatus under different light conditions. Despite the overall similarity, the complexes present in *Gemmatimonas phototrophica* and *Gemmatimonas groenlandica* have different absorption properties in the near-infrared region of the spectrum that make them more suitable for low or medium light, respectively. The main difference in absorption depends on the conformation of a single tryptophan residue that can form an H-bond with a neighboring bacteriochlorophyll. The presence or absence of this H-bond affects how the protein scaffold interacts with the bacteriochlorophylls, which in turn determines how light energy is transferred within and between the photosynthetic complexes.

## INTRODUCTION

Light absorption represents the initial step of photosynthesis. Phototrophic organisms have evolved different types of antenna systems optimized for their particular habitat. These antenna systems must cope not only with specific spectral irradiance in the particular habitat but also with changes in the light intensity. Too much light absorbed by the system can prove deleterious to the organism, e.g., by inducing the formation of harmful singlet-oxygen radicals, whereas too little light absorption does not provide sufficient energy for growth and places the organism at a competitive disadvantage. Therefore, phototrophic organisms have evolved the ability to match their light-harvesting (LH) capacity with the incident spectral irradiance. For instance, many cyanobacteria use chromatic adaptation to optimize the utilization of incident light (1).

Purple bacteria in the phylum Pseudomonadota (previously Proteobacteria) (2, 3) combine different adaptive mechanisms to flexibly match light-harvesting capacity with the incident light. The LH antenna complexes (LH2 and LH1) are housed in specialized intra-cytoplasmic membranes (ICM), and the cells contain much more ICM at low light (LL) than at high light (HL) (4). In addition, the number of photosynthetic units (PSUs) within the ICM increases under LL conditions (5, 6). Some species can produce a different type of LH2 at LL so that a steeper downhill energy transfer (ET) gradient is established within the PSU to funnel scarce excitons (a quantum of electronic excitation) into the reaction center (RC) (7–11). These different phenotypes are all controlled through complex transcriptional regulation of the responsible genes by light intensity (12).

The discovery of phototrophic species in the phylum Gemmatimonadota came as a surprise (13). The first species, *Gemmatimonas* (*Gem.*) *phototrophica* strain AP64, was isolated from a shallow freshwater lake in the Gobi Desert with phylogenetic evidence suggesting that it acquired the photosynthesis gene cluster (PGC) through horizontal gene transfer (HGT) from purple bacteria (14). Cryogenic electron microscopy (cryo-EM) determined the structure of its photosynthetic complex to 2.4 Å (15). The basic organization of its reaction center is analogous to purple bacteria; however, in contrast to any known species in Pseudomonadota, the RC is surrounded by two LH antenna rings. The inner ring is functionally and genetically similar to LH1 in purple bacteria, whereas the outer ring originates genetically from LH1 but optically resembles LH2. The outer ring antenna is called LHh (h for hybrid), and the resulting double-concentric ring RC-dLH complex is an effective and very stable structure (16). On the other hand, this arrangement confers certain limitations. As *Gem. phototrophica* synthesizes only complete RC-dLH complexes, there is no mechanism available to regulate the size or amount of the antenna when grown at different light intensities. Indeed, our recent transcriptomic study of *Gem. phototrophica* grown under different light conditions reveals very little change in the transcription patterns of not only the photosynthetic complexes but also the pigment and lipid biosynthetic genes that would be necessary to alter the amount of ICM or the number of PSU within it. This lack of any response strongly suggests that the adaptive mechanisms to changes in light intensity, described above in Pseudomonadota, simply do not occur in this species (17). Presumably, the HGT event that inserted the PGC into the genome of the early Gemmatimonadota cell then enabled some species to become phototrophic, but it did not confer any of the complex regulatory pathways and circuits to control it.

**Fig 1:**
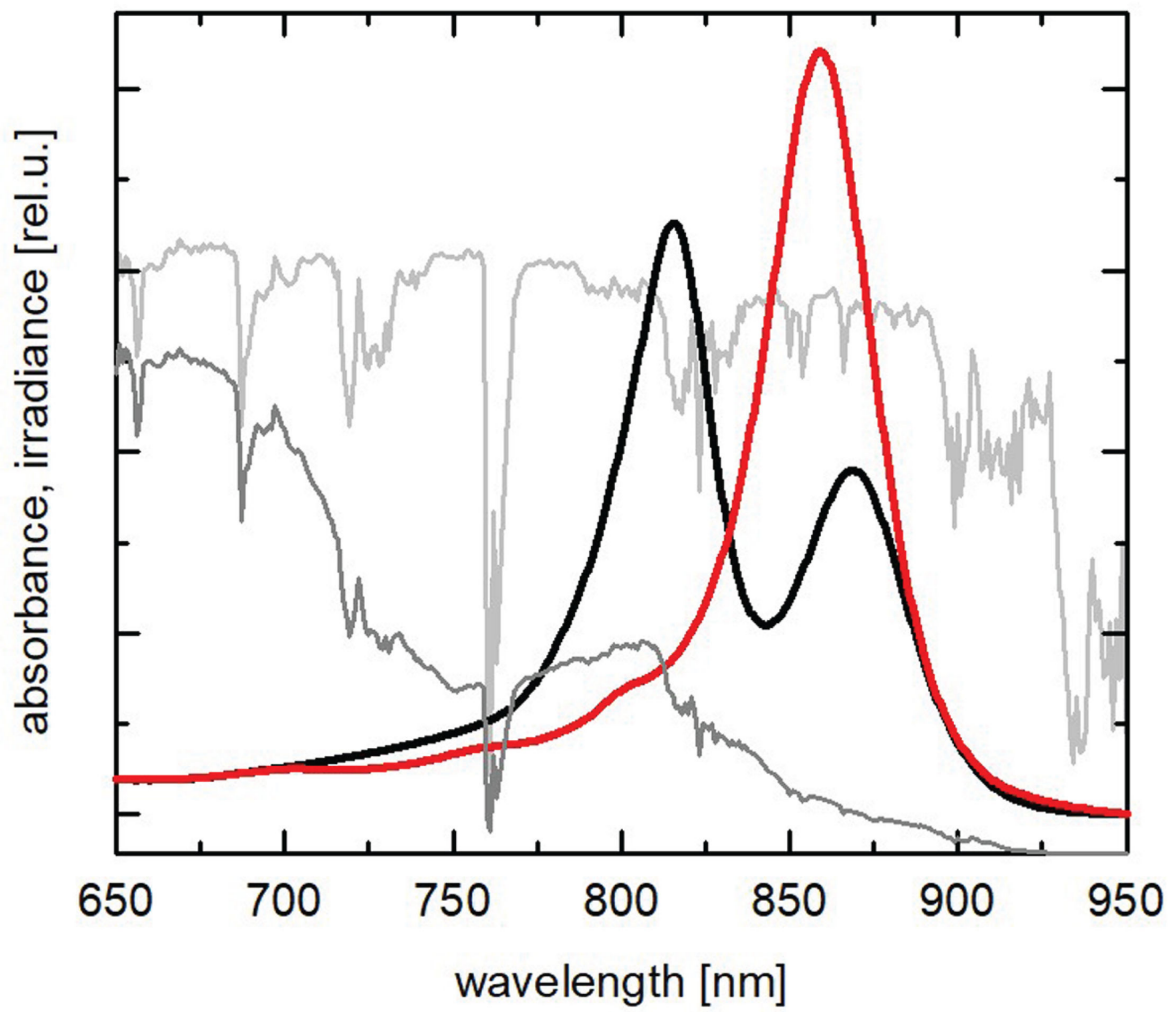
Absorption properties and water attenuation. Red and NIR part of the absorption spectra (area-normalized) of *Gem. phototrophica* (black) and *Gem. groenlandica* (red) with the incident solar spectrum (ASTM G173-03, light gray) and the solar spectrum attenuated by a 50 cm water column (dark gray). It is apparent that with increasing water depth, the *Gem. phototrophica* pigment composition offers superior spectral coverage.

A second phototrophic Gemmatimonadota species, *Gem. groenlandica* strain TET16, was isolated from a stream in Greenland (18). It also contains bacteriochlorophyll (BChl) *a*, but its absorption spectrum has only a single large absorbance band (maximum 860 nm) in the near-infrared (NIR) region, in contrast to two absorption bands (816 and 868 nm) in *Gem. phototrophica* (18). Irrespective of the molecular details that result in this difference, Fig. 1 illustrates that the *Gem. phototrophica* RC-dLH complex offers better absorption properties than *Gem. groenlandica* as light is attenuated by increasing water depth (19).

In order to understand the structural origin of this spectroscopic difference and the functional properties it exerts, the RC-dLH complex from *Gem. groenlandica* has been determined by cryo-EM and investigated using a range of biophysical and spectroscopic techniques. The results enabled a full structural and functional comparison of the two RC-dLH complexes, which in turn revealed the two phototrophic Gemmatimonadota species to have different LH capabilities through a combination of ongoing evolutionary pressure and a simple, yet completely novel effect induced by the rotamer conformation of a single amino acid residue.

## RESULTS

### Initial characterization of the *Gem. groenlandica* RC-dLH

**Fig 2:**
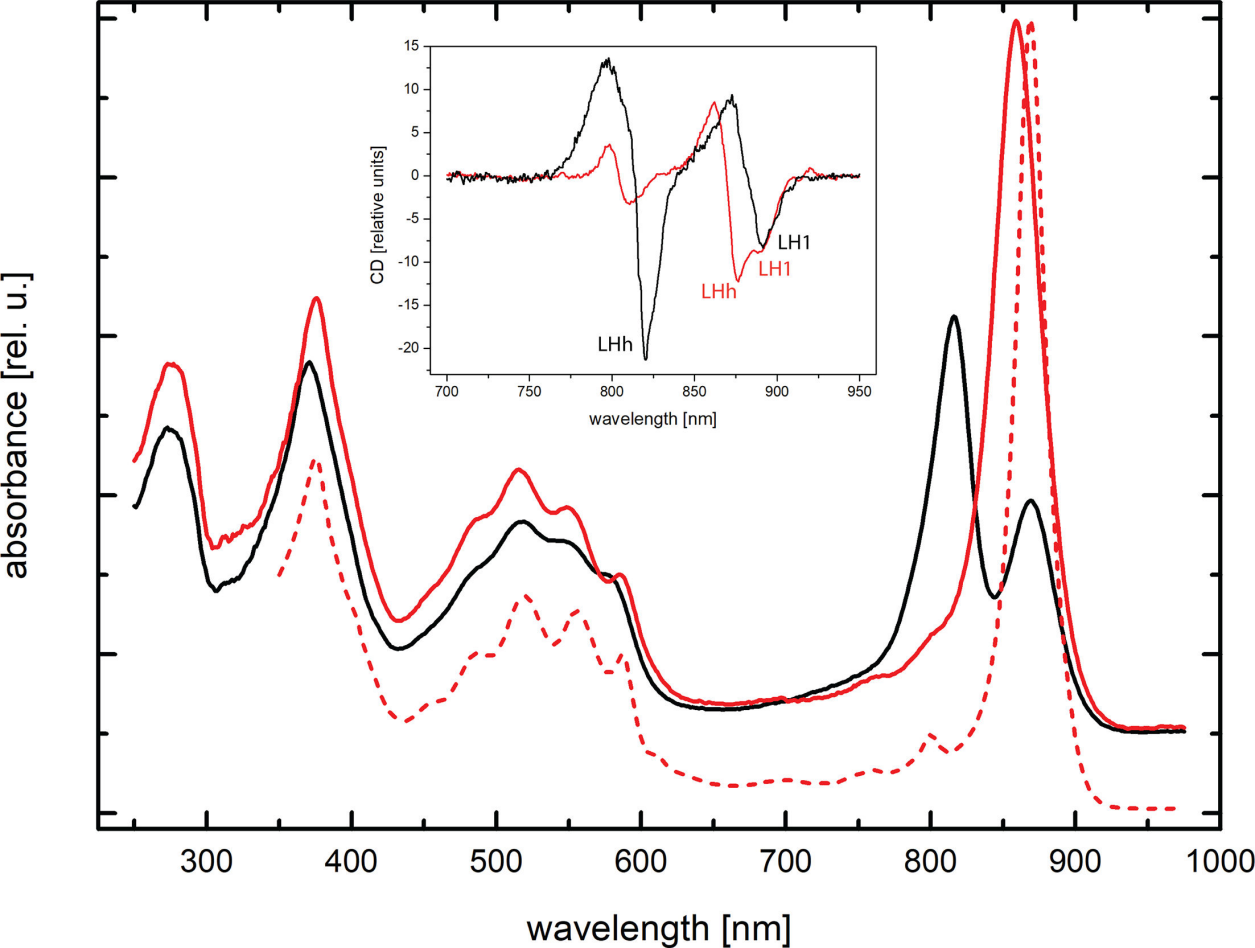
Spectroscopy of the purified RC-dLH complexes. RT absorption spectra of the RC-dLH complex from *Gem. groenlandica* (red line, NIR_max_ = 860 nm) and *Gem. phototrophica* (black line, NIR_max_ = 816 and 868 nm). Absorption spectrum at 77 K (red dash [offset]) of the purified *Gem. groenlandica* RC-dLH complex (NIR_max_ = 869 nm). The slight red shift of the 77 K NIR_max_ is consistently observed for these types of antenna complexes. The small peak at 800 nm originates from the RC. All spectra are normalized on the BChl Qx band at ~590 nm. Inset: 77 K CD spectra of the *Gem. groenlandica* and *Gem. phototrophica* complexes. As expected, the *Gem. phototrophica* complex has positive-negative features at ~814 and ~882 nm corresponding to LHh and LH1. The *Gem. groenlandica* complex has a single positive-negative feature at ~869 nm that splits in the negative region to reveal the presence of two antenna rings.

In *Gem. phototrophica*, the outer, LHh, ring gives rise to the 816 nm (B816) band in the absorption spectrum, and the inner, LH1, ring gives rise to the 868 nm band (B868). In this species, there is a third weakly bound BChl population that gives rise to the shoulder at ~800 nm (B800), further suggesting that LHh is functionally similar to LH2 in Pseudomonadota. In stark contrast, the purified RC-dLH complex from *Gem. groenlandica* exhibits only a single NIR absorption band with a maximum at 860 nm, Fig. 2. It appears that the LHh absorption is substantially red-shifted and overlaps with the LH1 absorption band. Interestingly, LHh and LH1 cannot be distinguished even in the 77 K spectrum, which still shows a single absorption band with NIR_max_ at 869 nm and a very minor shoulder on the red edge. The two antenna rings could only be differentiated in the 77 K CD spectrum (Fig. 2, inset).

High-performance liquid chromatography (HPLC) pigment analysis of the RC-dLH complexes purified from *Gem. groenlandica* revealed that almost 80% of BChl *a* had a phytol side chain, and a smaller amount had a geranylgeranyl side chain. In addition, the complex contained two main carotenoids: gemmatoxanthin (20) and a smaller amount of spirilloxanthin, Fig. S1. This is different from the *Gem. phototrophica* complex as it contains almost exclusively gemmatoxanthin (20). From the ratio of BChl *a* and bacteriopheophytin, it was estimated that an RC-dLH complex contains 81.6 ± 6.6 BChl *a* molecules, which is consistent with the presence of two antenna rings.

### Structural insights from the *Gem. groenlandica* RC-dLH

**Fig 3:**
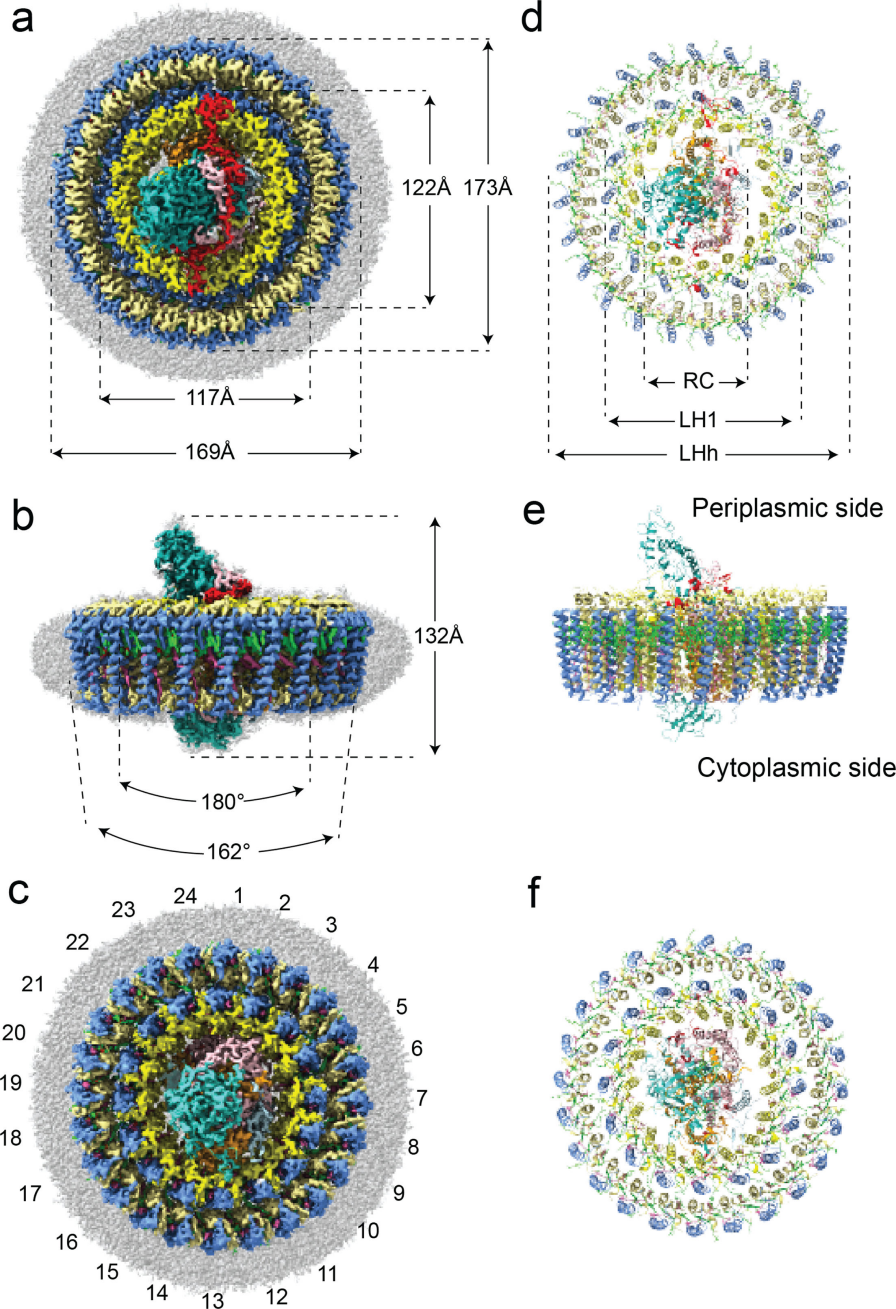
Cryo-EM structure of the RC-dLH complex from *Gem. groenlandica*. Views of the color-coded density maps (**a–c**) and corresponding ribbon presentations (**d–f**) of the RC-dLH Model-I structure. The color key is as follows: RC-C, sea green; RC-L, orange; RC-M, pink; RC-Hc, turquoise; RC Ht, light blue; RC-S, red; LH1 and LHh β-polypeptides cornflower blue; LH1 α1-polypeptides, yellow; LHh α2-polypeptides, khaki; BChl, lime; and gemmatoxanthin, pink. Panels **a and d** show periplasmic (top) views, panels **b and e** show membrane (side) views, and panels **c and f** are cytoplasmic (bottom) views. The distances given in panel **a** are the maximum and minimum distances of the ring ellipses. In the *Gem. phototrophica* RC-dLH complex, the extraneous polypeptide RC-S is present on the periplasmic face and RC-U on the cytoplasmic face. However, in *Gem. groenlandica,* this latter polypeptide was not detected even though the gene is present in the genome. The differences between Model-I and Model-II are shown in Fig. 4.

**Fig 4:**
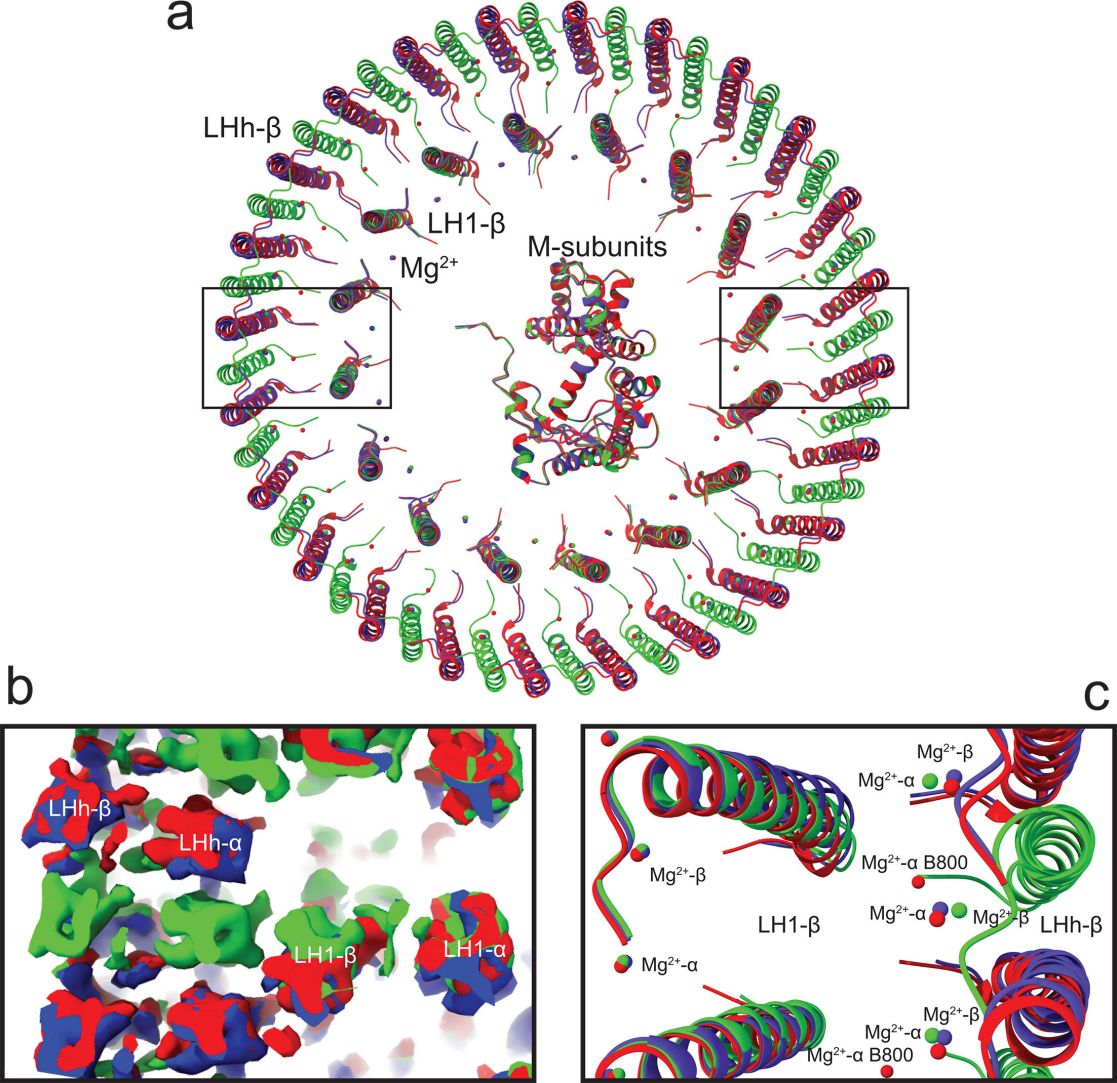
Superimposition of the RC-dLH structures. Red, *Gem. phototrophica*; blue, *Gem. groenlandica* Model-I; green, *Gem. phototrophica* Model-II. The two black rectangles on the left and right sides correspond to panels** b and c**, respectively. (**a**) The β-subunits in the rings of the three RC-dLH structures are shown (periplasmic side view) after superimposing the conserved RC M-subunit. The *Gem. phototrophica* and the *Gem. groenlandica* Model-I structures overlie each other easily and have almost identical dimensions. The *Gem. groenlandica* Model-I and Model-II overlie each other for RC-LH1, but the LHh ring is rotated clockwise by 7.5°. (**b**) A clipping slice of the contour density, in the plane of the membrane, through approximately the middle of the LHI and LHh transmembrane α-helices. Density for the former overlies each other in all three complexes, but the Model-II LHh α-helices in the latter do not. (**c**) An illustration to show how well the α-helices of the β-subunits overlie each other. The LH1 BChl Mg^2+^ also overlie each other, but the *Gem. phototrophica* and *Gem. groenlandica* LHh BChl Mg^2+^ are separated by approximately 1.3 Å.

In order to identify the differences between the two complexes, the structure of the *Gem. groenlandica* RC-dLH complex was solved by cryo-EM with a global resolution of 2.3 Å. Figure 3 reveals that it has a very similar overall architecture to that of *Gem. phototrophica* with two concentric antenna rings surrounding the central RC. The data processing workflow used to obtain the *Gem. groenlandica* RC-dLH particles produced two different classes of particles, Model-I and Model-II, as shown in Fig. S2. A superimposition of both models onto the *Gem. phototrophica* RC-dLH is shown in Fig. 4a and reveals that all complexes are slightly elliptical with the same overall dimensions. The *Gem. groenlandica* Model-I and Model-II RC and LH1 overlie each other, but the LHh ring is rotated clockwise by 7.5°. This is further illustrated by panels Fig. 4b and c. The map density is shown in Fig. 4b, and the respective differences in the positions of the BChl Mg^2+^ ions are shown in Fig. 4c. The Mg^2+^ ions from the LH1 ring BChl also superimpose well in this top (periplasmic) view; however, the Model-I and Model-II LHh BChl Mg^2+^ do not align and are translated from each other by ~1.3 Å. A close examination of the molecular interactions in the LH1 ring of the *Gem. groenlandica* and *Gem. phototrophica* complexes revealed minimal differences. A representative *Gem. groenlandica* LH1 αβ-heterodimer is shown in Fig. S3.

**Fig 5:**
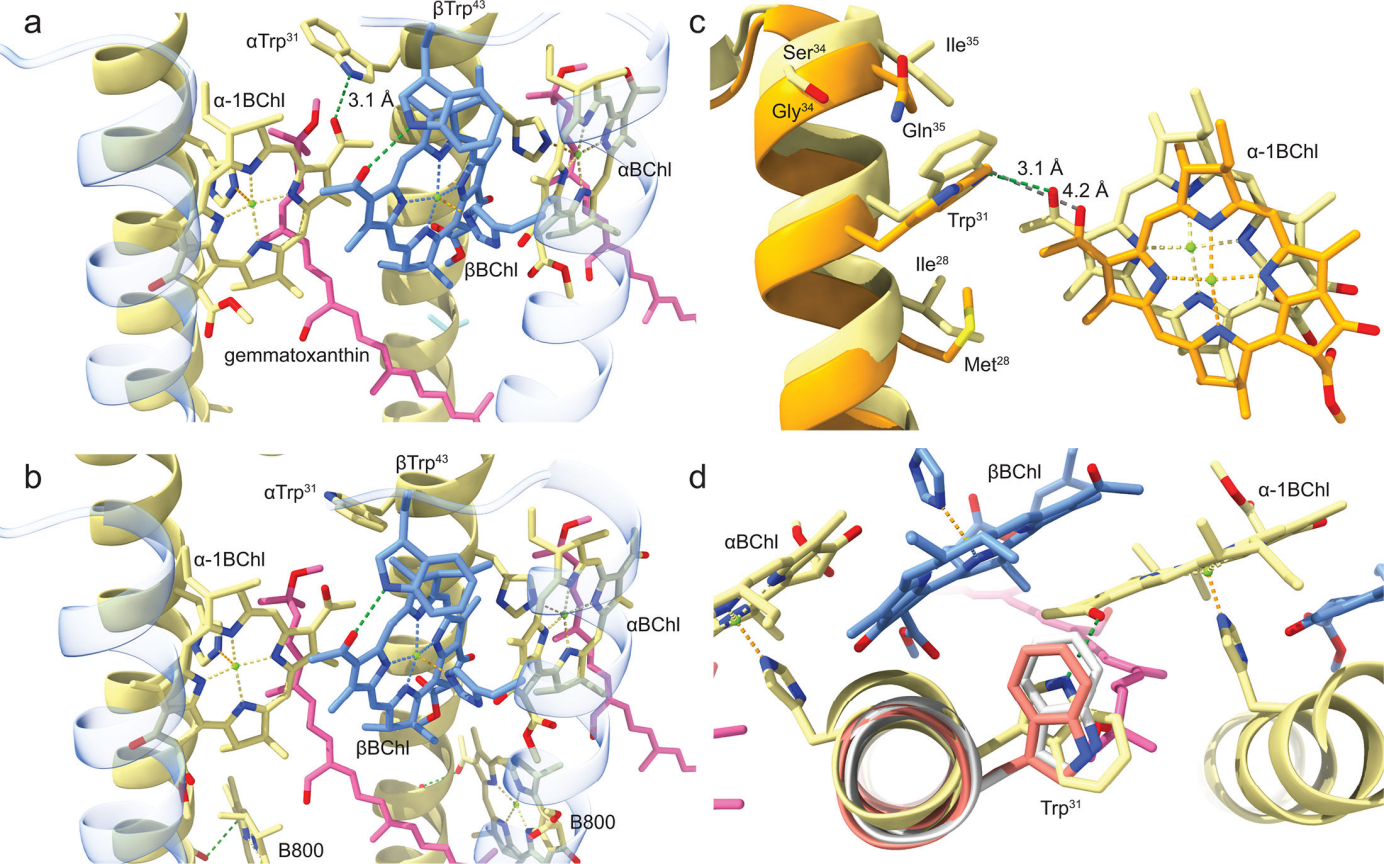
A comparison of both LHh ring αβ-heterodimers. (**a**) The two α-polypeptides and respective His-ligated BChls are colored khaki with the corresponding β-polypeptides and one His-ligated BChl in cornflower blue. The β-polypeptides are shown with 80% transparency for clarity. The gemmatoxanthin carotenoid is in pink, and, again for clarity, the BChl phytol tails have been removed. In the *Gem. groenlandica,* αβ-heterodimer αTrp^31^ has a rotamer conformation so that a 3.1 Å H-bond is able to form between αTrp^31^ and the α-1BChl C3^1^ keto group. (**b**) The *Gem. phototrophica* αTrp^31^ has a different rotamer conformation that makes H-bond formation to the α-1BChl C3^1^ keto group impossible. In this view, it can be seen that the B800 BChl lies almost perpendicular to the LHh α-BChl. (**c**) A view showing only an LHh α-polypeptide and its associated α-1BChl when the RC PufM polypeptide for the two RC-dLH complexes is superimposed (similar to Fig. 4). For ease of viewing, *Gem. groenlandica* is colored khaki and *Gem. phototrophica* in orange. It is apparent from this side view that *Gem. groenlandica* LHh is displaced slightly relative to *Gem. phototrophica* and that the distance (gray) between αTrp^31^ and the α-1BChl C3^1^ keto group in *Gem. phototrophica* (4.2 Å) is greater than the equivalent H-bond length in *Gem. phototrophica* (3.1 Å). The respective amino acid residues in the vicinity of αTrp^31^ are also shown, but these provide no clear explanation for the different αTrp^31^ rotamer conformations. (**d**) AlphaFold3 predictions for the α-LHh polypeptides (*Gem. groenlandica* in gray and *Gem. phototrophica* in salmon) aligned with the same polypeptide from the *Gem. groenlandica* structure. The *Gem. groenlandica* rotamer conformation prediction is the same as the *Gem. phototrophica* prediction and structure, but different from the *Gem. groenlandica* structure.

In both *Gem. phototrophica* and *Gem. groenlandica*, the central Mg^2+^ ions of LHh α- and β-BChl are bound to αHis^29^ and βHis^34^, respectively. All contacts supporting the LHh BChl ring are identical for both species, with the single exception that αTrp^31^ adopts a different rotamer conformation in each. The conformation adopted by the αTrp^31^ residue in *Gem. groenlandica* makes it possible for an H-bond to form, with an average length of 3.1 Å, to the α-1.BChl C3^1^ keto group. A *Gem. groenlandica* LHh heterodimer with the H-bond is shown in Fig. 5a. The equivalent heterodimer from *Gem. phototrophica* is given in Fig. 5b and shows that due to the different αTrp^31^ rotamer conformation, an equivalent H-bond is not possible. It is apparent from panels a and b of Fig. 5 that only amino acid residues on the αLHh α-helix turn facing the BChl C3^1^ keto group, above and below Trp^31^, are in sufficient proximity to affect the rotamer conformation. Therefore, Fig. 5c presents a side view of an αLHh α-helix from each species, with the residues shown that may influence Trp^31^ and the associated α-1BChl. This view was made by superimposing the RC PufM polypeptide to make it equivalent to Fig. 4. For ease of viewing, *Gem. groenlandica* is colored khaki and *Gem. phototrophica* in orange. In contrast to the top view shown in Fig. 4c, it is apparent from this side view that *Gem. groenlandica* LHh and its BChl ring are displaced relative to *Gem. phototrophica*. The Mg^2+^ to Mg^2+^ distance is ~1.6 Å, and the distance (gray) between αTrp^31^ and the α-1BChl C3^1^ keto group in *Gem. phototrophica* (4.2 Å) is greater than the equivalent H-bond length in *Gem. groenlandica* (3.1 Å). The respective amino acid residues in the vicinity of αTrp^31^ are also shown, but these provide no clear explanation for the different αTrp^31^ rotamer conformations. In *Gem. phototrophica*, there is no H-bond between Gln^35^ and Trp^31^. In order to determine if the primary sequence alone had an influence on the Trp^31^ rotamer conformation, AlphaFold 3 (21) was used to predict the structure for the two αLHh polypeptides. AlphaFold 3 predicted the *Gem. groenlandica* rotamer conformation is the same for both the *Gem. phototrophica* prediction and the structure (panel b) but different from that in the actual *Gem. groenlandica* structure (panel a). This suggests that the *Gem. groenlandica* primary sequence alone does not determine Trp^31^ rotamer conformation. The AlphaFold 3 αLHh prediction for each species is illustrated in Fig. 5d (*Gem. groenlandica* in gray and *Gem. phototrophica* in salmon), along with an αLHh polypeptide from the *Gem. groenlandica* structure.

**Fig 6:**
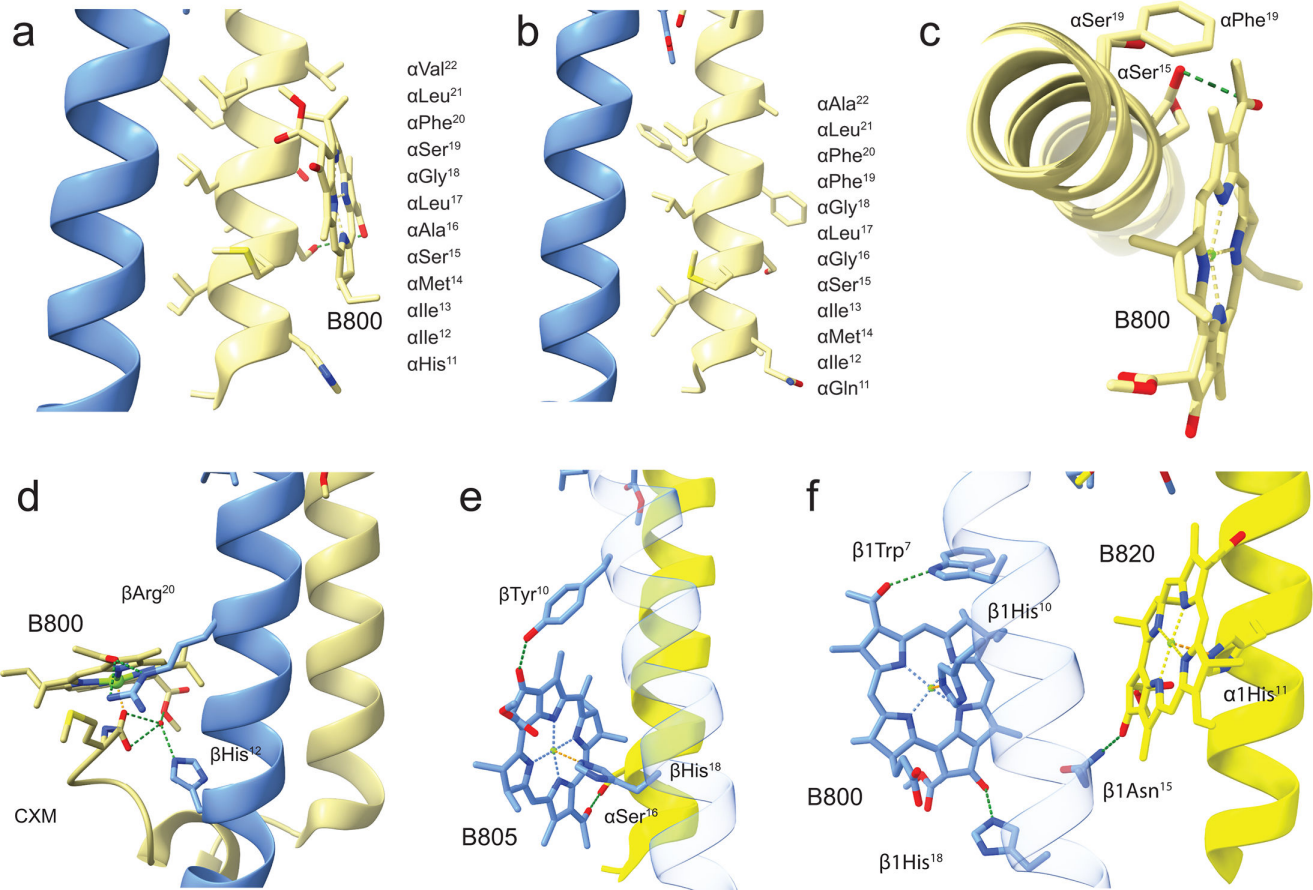
A comparison of B800 binding in different species. These views illustrate the varying levels of complexity through which B800 BChls are bound in different species of phototrophic bacteria. For orientation, a side view is given with the periplasmic side at the top. (**a**) *Gem. phototrophica* LHh α-polypeptide is oriented perpendicular to the plane of the membrane, with B800 weakly bound through hydrophobic interactions. The amino acids that form the α-helix in this region are shown, with the BChl stabilized by a weak 3.4 Å H-bond from αSer^15^ to the α-BChl C3^1^ keto group. None of the other amino acid side chains on the helix appear to play a direct role in the binding. (**b**) *Gem. groenlandica* LHh does not contain a B800 population, and the αSer^15^ side chain is oriented such that an H-bond is not possible. The amino acids that form the α-helix are shown. (**c**) A view perpendicular to the membrane looking down a superimposed LHh α-helix of *Gem. phototrophica* and *Gem. groenlandica,* clipped in the region of the B800. The αSer^15^ side chain in the latter species is oriented such that it is not able to form an H-bond to the α-BChl C3^1^ keto group. It is also seen that αPhe^19^ in *Gem. groenlandica* does not impede the binding of BChl through any steric hindrance. (**d**) The *Rbl. acidophilus* strain 10050 LH2 B800 is bound by an α-polypeptide N-terminal carboxymethionine group (CXM). Typical for nonameric LH2 complexes in Pseudomonadota, the B800 bacteriochlorin macrocycle ring is oriented in the plane of the membrane. These polypeptides are transcribed from the *puc*BA genes. (**e**) The B805 from *Rof. castenholzii* is also oriented perpendicular to the membrane normal with the B805 held in place by a β-His, rather than an α-His in Pseudomonadota. The β-polypeptide is shown with 80% transparency for ease of viewing, and these polypeptides are transcribed from the *puf*BA genes. (**f**) A simplified representation of the B800/B820 binding site from *Hlr. halochloris* showing that both bacteriochlorin macrocycle rings are also positioned perpendicular to the membrane normal. The β-polypeptide is shown with 80% transparency for ease of viewing.

A comparison of B800 binding in LHh is presented in Fig. 6. The *Gem. phototrophica* RC-dLH B800 in Fig. 6a is not bound through an interaction of the central Mg^2+^ ion with a His side chain, rather BChl is stabilized by a weak 3.4 Å H-bond from αSer^15^ to the α-BChl C3^1^ keto group and through weak hydrophobic interactions with the bacteriochlorin ring oriented perpendicular to the plane of the membrane. In contrast, the LHh ring of *Gem. groenlandica* does not contain B800 pigments, Fig. 6b, as αSer^15^ also adopts a different rotamer conformation, which prevents the formation of the H-bond to the α-BChl C3^1^ keto group. In both Fig. 6a and b, the different hydrophobic residues in this part of the alpha-helix are listed and shown. An obvious difference is that αSer^19^ in *Gem. phototrophica* is replaced by the bulky αPhe^19^ in *Gem. groenlandica*. The different αSer^15^ orientations are confirmed by superimposing the α-alpha helices from both species in Fig. 6c, which also shows that the lack of B800 in this species is not due to steric hindrance from αPhe^19^. The full superimposition of the *Gem. phototrophica* and *Gem. groenlandica* polypeptides is provided in Fig. S4. The presence of B800 in *Gem. phototrophica* gives this LHh ring functional commonality with LH2; therefore, the B800 binding pocket from a typical Alphaproteobacterial LH2, *Rhodoblastus* (*Rbl.*) *acidophilus* strain 10050 (PDB: 1NKZ), is shown in Fig. 6d. This shows that LH2 B800 binding is rather different from LHh B800 as a number of different bonding interactions hold the BChl in place in the plane of the membrane (22). The non-chlorosome-containing *Roseiflexus* (*Rof.*) *castenholzii* from the family Chloroflexaceae has an RC-LH complex (PDB: 8IUG) that binds a B800-like population (B805) with the bacteriochlorin ring also oriented perpendicular to the plane of the membrane (Fig. 6e). In this case, however, the central Mg^2+^ is bound to His^18^ on the β-polypeptide (23). The most complex of all with respect to B800 is the RC-LH1 complex from the extremophilic Gammaproteobacteria *Halorhodospira* (*Hlr.*) *halochloris* (PDB: 8K5O) (24). This complex contains α-, β-, and γ-polypeptides and two different B800-like BChl *b* populations, with each population having the central Mg^2+^ coordinated to a histidine side chain: B800 BChl Mg^2+^ is bound to a β1His^10^ and the B820 BChl Mg^2+^ to α1His^11^ (Fig. 6f).

### Light-harvesting properties of the RC-dLH complexes

**Fig 7:**
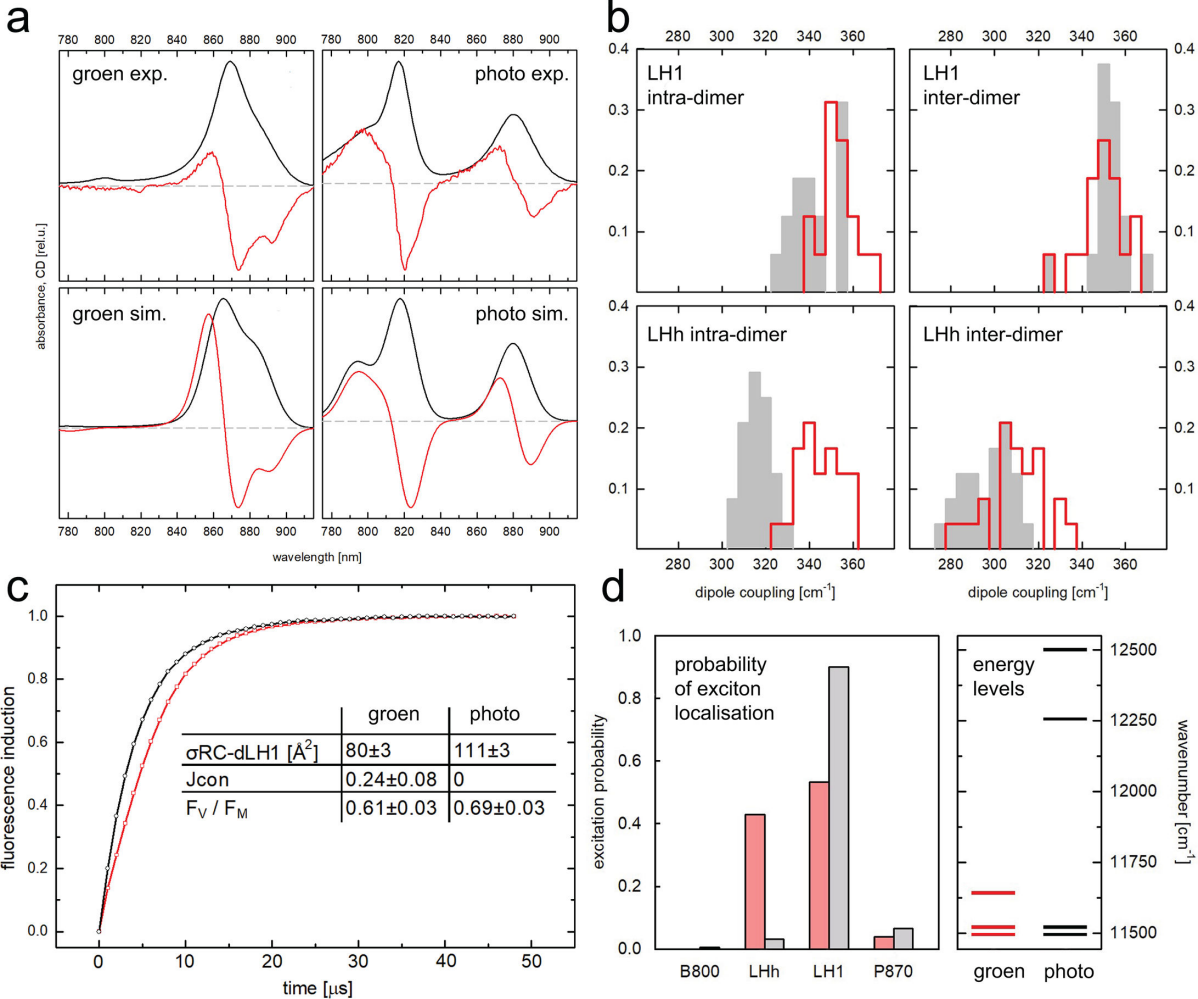
Biophysical analyses of the RC-dLH complexes. For *Gem. groenlandica* and *Gem. phototrophica*, the abbreviations groen and photo have been used, respectively (**a**) Spectra simulations using the point-dipole approximation. The absorption spectra are given in black and the CD spectra in red. (**b**) Overview of excitonic coupling (in cm^−1^) between α and β BChls in the RC-dLH complex of *Gem. phototrophica* and *Gem. groenlandica* separated into the coupling within the pair of pigments belonging to the same two-helix-two-BChl subunit of the antenna: intra-dimer and coupling between the neighboring subunits: inter-dimer. The two top-row panels represent the inner, LH1 part of the complex, whereas the two bottom-row panels correspond to the outer, LHh ring. (**c**) Single-turnover BChl *a* fluorescence induction kinetics and the derived parameters describing the RC-dLH photochemical activity of the two strains, respectively: functional antenna cross-section σ_RC-dLH1_, antenna connectivity J, and maximal photochemical yield *F*_*V*_/*F*_*M*_. (**d**) Probability of excitation localization (at 280 K) on the different BChl populations of the RC-dLH from *Gem. groenlandica* and *Gem. phototrophica*, assuming that the system is in thermodynamic (Boltzmann) equilibrium. The right panel shows the energy levels (represented as wavenumber, cm^−1^) of these respective pigment populations belonging to the spectroscopic subunits of the complex.

Biophysical experiments were conducted to better understand the underlying reasons for the different *Gem. phototrophica* and *Gem. groenlandica* NIR spectra and how this relates both to the presence or absence of the LHh αTrp^31^ H-bond and the overall effect on LH. The experimental results are shown in Fig. 7.

Pigment couplings of both RC-dLH complexes were computed between BChls and used to simulate the steady-state, optical absorption, and CD spectra, Fig. 7a. For the sake of simplicity, the treatment was limited to the point-dipole approximation using only the Qy transition dipoles, and any higher excited states were neglected. It is evident that the steady-state spectroscopic properties of the *Gem. groenlandica* complex proved to be challenging for the present simulation model, even if the overall shape of the spectra can be reproduced with reasonable quality. In particular, the non-conservative shape of the CD band of the LHh ring is not reproduced well. This is often the case in these simulations due to the neglect of the coupling of Qy to the higher excited states. It is worth mentioning that excitonic interactions alone were not sufficient to change the absorption band at ~816 nm of *Gem. phototrophica* LHh to the 860 nm peak of *Gem. groenlandica*. The interactions necessary to generate the proper shape of the CD spectrum of B816 are due to the B800 pigment. Indeed, removal of this transition dipole from the model yielded a CD spectrum consisting of a pair of (almost) conservative bands at 816 and 868 nm. In order to achieve the proper position of the LHh spectrum, the necessary shift of the site energy of the BChl is in the order of hundreds of wavenumbers, going from *Gem. phototrophica* to *Gem. groenlandica*. This most likely indicates the necessity to invoke an effect not accounted for by the modeling approach used, such as charge-transfer states. These are very sensitive to slight changes in inter-pigment geometry. While the present approach cannot deal with charge-transfer states *per se*, the computed dipole couplings from the simulations integrate the positional information on the interacting dipoles, including both distance information (through the r^−3^ dependence) and orientation (through the dot product of the transition dipole vectors). They can still serve as a sensitive indicator of the overall structural differences between the complexes. In contrast, the *Gem. phototrophica* complex is adequately captured, including the shape of the CD signal in the region around 800 nm, with the asymmetry of the CD signals. In *Gem. phototrophica*, all of the necessary contributions that shift the position of the maximum of the spectral band, with respect to the site energy of the pigment, can be ascribed to the excitonic interaction only.

In Fig. 7b, the computed coupling is shown between α and β pigments in both the LH1 and LHh, separated into intra-dimer and inter-dimer interactions. It is readily observed that the feature in which the two complexes differ significantly is the intra-dimer coupling between α and β BChl within a single LHh subunit (dimer), which is enhanced in *Gem. groenlandica* by about 10% compared to *Gem. phototrophica*. It can be estimated that about 50% contribution to this difference comes from a decreased distance between the α- and β-pigments. This can also be expected to affect the propensity to form charge-transfer states.

In order to assess the functional differences between the two RC-dLH complexes, photochemical efficiency was measured in whole cells by single-turnover BChl *a* fluorescence induction kinetics (Fig. 7c). This experiment provides information about the maximum photochemical yield *F_V_/F_M_* and functional cross-section σ. In addition, the exciton connectivity parameter *J* can be extracted from the shape of the measured curve (25). An exponential rise to the maximum kinetics indicates no energy transfer from one complex to another (i.e., no connectivity). When there is inter-complex energy transfer, if the exciton first arrives at a closed RC, it can jump to a different complex with an open RC instead, and the fluorescence rise will be more sigmoidal. The maximal photochemical yield *F*_*V*_*/F*_*M*_ and the functional antenna cross-section (σRC-dLH) are about 12% and 30% larger, respectively, in *Gem. phototrophica* than in *Gem. groenlandica*. The most prominent difference between the strains was the sigmoidal fluorescence induction curve in *Gem. groenlandica* (Fig. 7c, red), which documents the excitonic exchange (connectivity) among the *Gem. groenlandica* RC-dLH. In contrast, the fluorescence induction curve of *Gem. phototrophica* follows an exponential rise to maximum kinetics (Fig. 7c, black), confirming the absence of exciton exchange reported before (26).

The RC-dLH complex functions by transferring excitons from the LHh ring to LH1 and on to the RC to initiate photochemistry. The probability of excitation localization (at 280 K) on the different BChl populations of the RC-dLH was calculated, Fig. 7d, from *Gem. groenlandica* (red) and *Gem. phototrophica* (gray/black), assuming that the system is in thermodynamic (Boltzmann) equilibrium. The right panel in Fig. 7d shows the energy levels (represented as wavenumber, cm^−1^) of the respective BChl populations in the spectroscopic subunits of the complex. It is apparent that the probability of excitation localization on the LHh and LH1 rings of *Gem. groenlandica* (red) is 45:55, respectively (the LHh ring in this complex has no B800 population), whereas in the *Gem. phototrophica* RC-dLH, the probabilities are 1 (B800): 4 (LHh): 95 (LH1). Thus, *Gem. phototrophica* directs excitation energy to LH1, which is explained by the large energy gap between the LHh and LH1 that restricts the energy back transfer.

### Origin of the LHh ring

**Fig 8:**
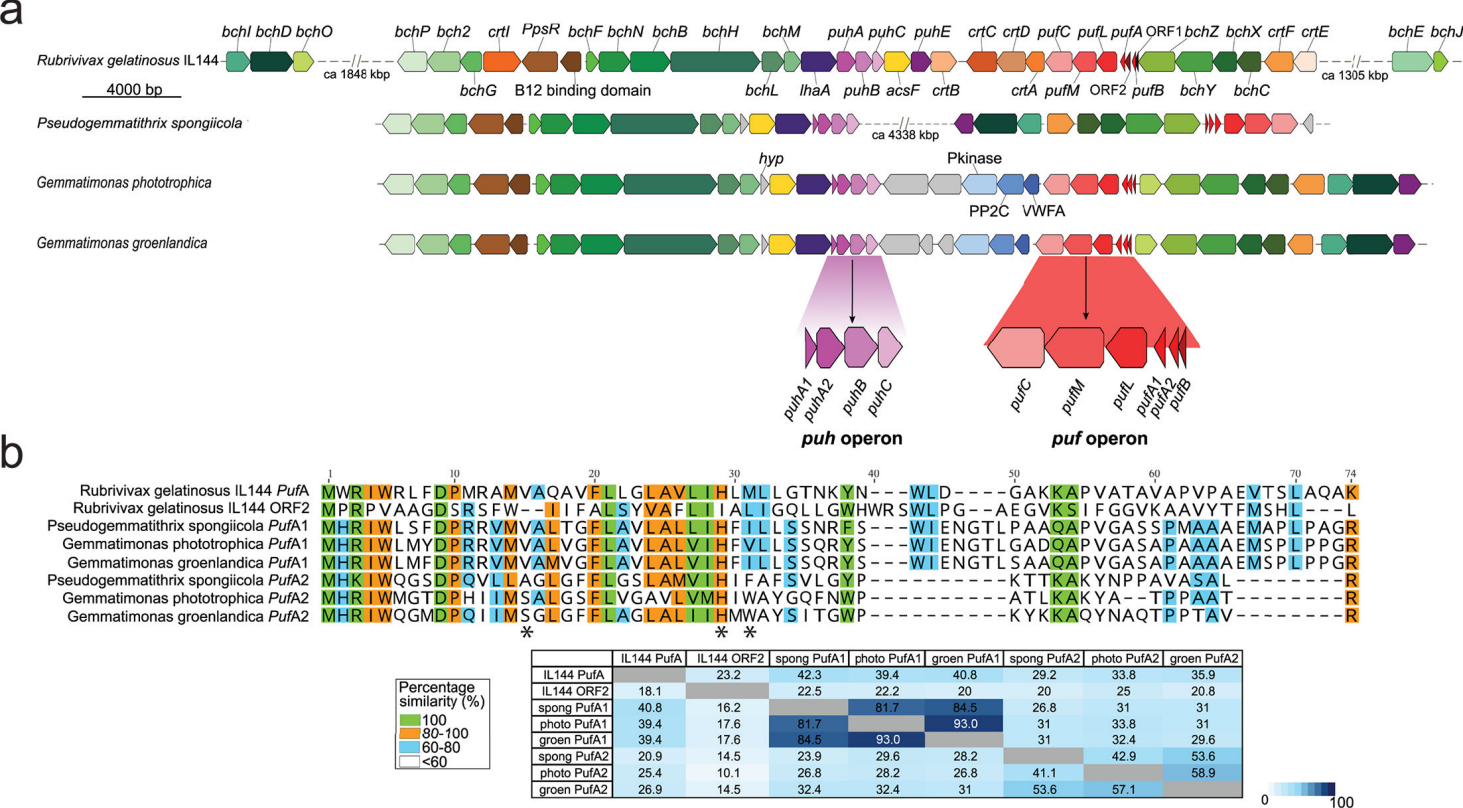
Gemmatimonadota PGC comparison. (**a**) *Pgt. spongiicola*, *Gem. phototrophica,* and *Gem. groenlandica* compared to *Rvi. gelatinosus* (Pseudomonadota). Both *Rvi. gelatinosus* and *Pgx. spongiicola* have the PGC separated by a large insert of non-phototrophic genes that have been omitted from the figure, but the distance between the genes is provided. Different shades of color are used to indicate the different gene groups within the PGC: green, *bch* genes involved in BChl biosynthesis; red, *puf* operon encoding the RC; purple, *puh* operon; orange, *crt* carotenoid biosynthesis genes; brown, *pps*R gene and B12 binding domain; yellow, *acsF* (Mg-protoporphyrin IX monomethyl ester aerobic cyclase); blue, genes not involved in photosynthesis; and gray, hypothetical genes. Additionally, the presence of the two *puf*A genes (*puf*A1 and *puf*A2) and the split of the *puh*A gene (*puh*A1 and *puh*A2) is detailed below the *Gem. groenlandica* PGC in purple and red, respectively. (**b**) Polypeptide alignments of the putative *Pgt. spongiicola*, *Gem. phototrophica,* and *Gem. groenlandica* PufA1 and PufA2 polypeptides with PufA and ORF2 from *Rvi. gelatinosus* IL144. For brevity, the abbreviations groen and photo have been used, respectively, for *Gem. groenlandica* and *Gem. phototrophica*. The table for each alignment shows the percentage identities for each sequence. The left side is percentage identities computed by multiple sequence alignment, and the right side shows the percentage of pairwise identities. A color-coded legend is shown beside each table, with white representing the lowest identity and dark blue the highest. A color-coded key for the residue percentage similarity is also given. Three amino acids are marked with asterisks: αSer^15^ that forms the only H-bond to the B800 in *Gem. phototrophica* (Fig. 6c), αHis^29^ binds the central Mg^2+^ ion of the α-BChls in the LHh ring, and αTrp^31^ that adopts a different rotamer conformation between *Gem. phototrophica* and *Gem. groenlandica* (Fig. 5).

The PGCs of *Gem. groenlandica* and *Gem. phototrophica* are shown in Fig. 8a, along with *Pseudogemmatithrix* (*Pgt.*) *spongiicola* (27) and the Pseudomonadota species *Rubrivivax* (*Rvi.*) *gelatinosus* for comparison. The original donor species for the HGT event that conferred phototrophy on the Gemmatimonadota is not known; however, the *Gem. groenlandica* and *Gem. phototrophica* PGCs bear a close resemblance to the PGC in modern *Rvi. gelatinosus*. This suggests that the donor in the HGT event may have been an ancient purple bacterium related to this species (14).

In both the *Gem. phototrophica* and *Gem. groenlandica* RC-dLH complexes, the LH1 ring is composed of 16 repeating heterodimeric αβ-units, encoded by the *puf*BA1 genes and also contains two BChl and the carotenoid gemmatoxanthin (20). The *puf*BA2 genes allowed *Gemmatimonas* to evolve an outer LHh ring surrounding the central RC-LH1 complex composed of 24 repeating heterodimeric αβ-units (16). The outer ring shares the same β-subunit with the inner ring but has its own α-subunit (encoded by the *puf*A2 gene). Unlike most other species of Pseudomonadota, the *puf* operon in *Rvi. gelatinosus* does not only contain pufBALMC genes (*puf*BA encode the LH1 ring β- and α-polypeptides and *puf*LMC encode RC L-, M-, and C-polypeptides, respectively); rather, there are also two open-reading frames (ORFs) of unknown function. ORF1 is directly upstream of *puf*B, and ORF2 is upstream of *puf*A (28). This proximity suggests the possibility that in the phototrophic Gemmatimonadota species, *puf*A1 originated from *puf*A in the ancient *Rvi. gelatinosus* and *puf*A2 originated from ORF2. Therefore, PufA and the putative *Rvi. gelatinosus* ORF2 polypeptide were aligned with the three PufA1s and the three PufA2s from the Gemmatimonadota species. The result is shown in Fig. 8b. The low multiple sequence identity between ORF2 and PufA2 (14.5%, 10.1%, and 14.5%), as well as the lack of the conserved, central α-BChl Mg^2+^ binding His at position 29, suggests that the *puf*A2 gene did not originate from ORF2. The *Rvi. gelatinosus puf*A gene has greater multiple sequence identity with *puf*A1 from Gemmatimonadota (40.8%, 39.4%, and 39.4%) but less with *puf*A2 (20.9%, 25.4%, and 26.9%). This suggests that *puf*A from the ancient donor species became, after the HGT event, *puf*A1 in Gemmatimonadota. Later, *puf*A1 underwent gene duplication to form *puf*A2. The latter gene then diverged from *puf*A1 and evolved to allow the formation of LHh, with both ORF1 and ORF2 at some point being deleted. Another modification specific to Gemmatimonadota is that the *puh*A gene is split into *puh*A1 and *puh*A2. This gene is responsible for the RC H-subunit in Pseudomonadota, and the effect of this mutation on the *Gem. phototrophica* RC structure is shown in Fig. S5. It can be reasonably speculated that it was only after this mutation that the RC could be assembled by the rudimentary cellular machinery. As the *puf*A2 and split *puh*A genotypes are present in both *Gemmatimonas* and *Pseudogemmatithrix*, it follows that they originated relatively recently after the HGT event and before divergence into distinct genera. For comparison, the alignments of PufB and PufM are presented in Fig. S6.

## DISCUSSION

The main finding of this work is that there is now a comprehensive explanation of how two different phototrophic *Gemmatimonas* strains have adapted their LH apparatus to grow under different light conditions. The overall structural morphology of the RC-dLH complex and the basic protein-protein and protein-pigment interactions of the LH1 ring are essentially identical in both *Gem. groenlandica* and *Gem. phototrophica*. The drastic difference between the NIR absorption spectra of each of the RC-dLH complexes is caused by two modifications within LHh.

The first is that a single amino acid residue, αTrp^31^, in LHh adopts a different rotamer conformation in each complex. Due to its unique properties, tryptophan is able to adopt many different roles in proteins (29), e.g., rotation of the tryptophan side chain is involved in the photo-activation of the Blue Light Using Flavin (BLUF) protein. In the dark-adapted BLUF, the tryptophan residue points away from the flavin and can move freely. Upon blue-light excitation, the tryptophan moves closer to the flavin and forms a hydrogen-bond network around the flavin molecule (30). In *Gem. groenlandica*, a 3.1 Å H-bond is formed between α-Trp^31^ and the C3^1^ keto group of the previous heterodimer α-BChl (Fig. 5a and c). *Gem. phototrophica* LHh also contains α-Trp^31^, but the aromatic side chain adopts a different conformation so that the H-bond is not able to form (Fig. 5b and c). H-bonds are already well known to modulate absorption bands in Pseudomonadota, but these absorption changes are caused by variations of two specific amino acids in heterologous primary sequences of the antenna subunit (31–34). This Trp^31^ H-bond to BChl now provides the explanation for both the stronger LHh inter-dimer excitonic coupling in *Gem. phototrophica* than *Gem. groenlandica* (Fig. 7c) and for the resulting effects on excitonic migration within the complex. In *Gem. phototrophica*, an exciton on LHh (B816) is rapidly transferred to LH1 (B868) due to the large difference in energy levels between the two bands (26), but the reverse process (“uphill” from LH1 to LHh to another LHh) is unlikely (Fig. 7d). This explains the absence of excitonic connectivity between the complexes in this species (Fig. 7b). This situation is advantageous at lower-light intensities as the “scarce” exciton is directly funneled into the LH1 ring. In contrast, in *Gem. groenlandica,* the difference in energy levels for ET between LHh (~860) and LH1 (~868 nm) is rather minimal (Fig. 7d) and so the exciton can migrate “uphill” from LH1 to LHh in *Gem. groenlandica* and even move to another RC-dLH complex to find an “open” RC. Such connectivity increases trapping efficiency under higher-light conditions when some of the RC are already “closed.”

Figure 6c shows that in the *Gem. phototrophica* RC-dLH, the B800 BChl is stabilized by only a single H-bond from αSer^15^ to the α-BChl C3^1^ keto group and through weak hydrophobic interactions. It is oriented perpendicular to the plane of the membrane. *Gem. groenlandica* RC-dLH does not contain the B800 BChl population, primarily because αSer^15^ adopts a different conformation and cannot form an H-bond. In contrast, the B800 binding pockets in *Rbl. acidophilus*, *Rof. castenholzii,* and *Hlr. halochoris* all have a number of interconnected H-bonds that hold the BChl in place in the plane of the membrane. The orientation B800 porphyrin ring with respect to the membrane is not important *per se* as long as the Qy dipole remains optimally aligned for energy transfer. However, for *Gem. phototrophica*, the B800 orientation may have important consequences for energy transfer as the Qy dipole appears to be nearly perpendicular between B800 and B816. The ET rate was measured at 0.4 ps but was described as sub-picosecond to picosecond range due to the error incurred and because the actual bleaching of B800 was not visible (15). The direct excitation of B816 and B868 through the upper exciton level may have obscured the dynamics to some extent. The coupling between B800 and B816 in *Gem. phototrophica*, with the parameters used to model the spectra (Fig. 7a), is of the order of 1 cm^−1^ (up to 10 cm^−1^), and most likely, ET is not to an individual BChl but to the excitonic states of the coupled B816 BChls. Assuming that the emission of the B800 BChl has a similar Stokes shift as BChl in solution (in the range of 200–250/cm), then for absorption at 800 nm, the emission will be at 810–820 nm, i.e., directly into the B816 band. Therefore, although the *Gem. phototrophica* B800 binding site is rather weak and the orientation unfavorable for ET, it is functional and increases the light-harvesting capability of the RC-dLH complex under low-light conditions, thereby accounting for the increased optical antenna size for the *Gem. phototrophica* RC-dLH complex measured in Fig. 7b.

In conclusion, the large B816 (including B800) LHh absorption band and the energetic separation to B868 allow *Gem. phototrophica* to better utilize light at greater depths underwater. This is consistent with the fact that *Gem. phototrophica* prefers growth under lower light and lower oxygen concentrations, common in deeper parts of the water column (17). In *Gem. groenlandica*, the αTrp^31^-induced H-bond red-shifts the LHh absorption band to ~B860, causing it to overlap with the LH1 absorption, resulting in a single, combined band at ~860 nm. This results in the RC-dLH complexes being substantially more iso-energetic in the membrane, which may be advantageous under higher irradiance and oxic conditions (18, 35). Using these two RC-dLH structures, further studies are now possible using quantum mechanics to dissect the excitonic states and site energies of intra and inter-dimer coupling in RC-dLH, as well as establishing the precise role of (the extremely distance-dependent) charge-transfer states in the *Gem. groenlandica* LHh absorbance shifts.

## MATERIALS AND METHODS

### RC-dLH complex purification

Cells of *Gem. groenlandica* were grown with stirring and constant aeration as previously described (18). The RC-dLH complex was initially purified, essentially, as previously described for *Gem. phototrophica* (15). However, the previous solubilization conditions (2.0% *n*-dodecyl-β-D-maltoside [DDM] and 0.2% Triton X-100, 1 h) resulted in the formation of multiple-orientation dimeric complexes that were unsuitable for cryo-EM analysis. The solubilization conditions were subsequently amended by using progressively lower detergent concentrations. The condition finally discovered that produced monomer complexes was 1% DDM for 5 s. Pigments were extracted from the RC-dLH complex using 100% methanol and analyzed by the HPLC as previously described (20).

### Spectroscopy and modeling

Absorption spectra were recorded using a UV2600 (Shimadzu, Japan) spectrometer with an integrating sphere. Circular dichroism was measured with a Jasco J-715 instrument equipped with an infrared-extended detector, and the detection bandwidth for near-infrared CD measurement was 5 nm. Low-temperature measurements were performed using a nitrogen bath cryostat (OptistatDN, Oxford Instruments, UK) on samples supplied with 60% (vol/vol) glycerol/buffer mixtures to ensure the formation of homogeneous glass upon cooling. Kinetics of BChl fluorescence were measured using FL-3000 fluorometer (Photon Systems Instruments Ltd., Czech Republic) as previously described (36).

Modeling of optical spectra was done using the exciton Hamiltonian as in reference 16 with inter-pigment couplings computed using the point-dipole approximation (using the code found at https://github.com/dbina/CDC). Values of the transition dipole moment were 5.8 D for all pigments (effective value, setting the dielectric constant to 1). To minimize the number of free model parameters, we choose to use the same value of inhomogeneous broadening (diagonal disorder), 440 cm^−1^, for all pigments, and the stick spectra of the excitonic states were given the same homogeneous broadening width of 220 cm^−1^. While, as shown in references 37 and 38, better fits of experimental spectra can be achieved when differences in dipole moments and broadening parameters between pigment pools are allowed, it is at the cost of increasing the set of free parameters. The site energies of the pigments were adjusted manually to give satisfactory agreement of simulated and experimental spectra. The values were (expressed in wavelength units, nm; pool: α/β) as follows: *Gem. phototrophica*, B868: 816/819; B816: 770/770; and B800: 802; *Gem. groenlandica*, LH1: 820/820; LHh: 807/807.

### Cryo-EM sample preparation and data collection

Quantifoil carbon Au 300-mesh grids (Quantifoil Micro Tools GmbH) were glow discharged in residual air for 60 s using GloQube (Quorum Technologies). A volume of 3 µL of purified *Gem. groenlandica* RC-dLH solution (OD at Qy = 114) was applied to the carbon side of the grid and blotted, then plunge-frozen into liquid ethane using a Vitrobot Mark IV (Thermo Fisher Scientific) operating at 4°C and 100% humidity, with a blot time of 3 s and a blot force of −3. Grids were stored in liquid nitrogen until required. Electron micrograph movies were collected using a Titan Krios (Thermo Fisher Scientific) equipped with a Falcon 4i direct electron detector operating at 300 kV with Selectris X energy filter mode with a 10 eV slit. Data sets were collected via EPU using aberration-free image shift with fringe-free illumination. Micrograph movies were collected at a nominal magnification of 165,000×, corresponding to a pixel size of 0.732 Å at the specimen level. Full data collection details for each data set are provided in Table 1.

**TABLE 1:** Cryo-EM data acquisition, model refinement, and validation statistics

Protein source	Photosynthetic bacterium
Data collection and processing	
Protein sample	RC-dLH Model-I/RC-dLH Model-II
Microscope	ThermoFisher Titan Krios G3i
Voltage (kV)	300
Camera	Falcon F4
Energy filter	Yes
Energy filter slit width	10 eV
Magnification	165,000×
Defocus range (μm)	−0.8 to −2.4
Pixel size (Å)	0.732
Electron fluence (e^−^/Å^2^)	60.00
Exposure time (s/frame)	0.13
Electron fluence per frame (e/Å^2^/frame)	1.5
Number of frames per movie	40
Number of movies acquired	23,012
Number of movies used	22,800
Initial no. particle images	935,540
Model label	RC-dLH-I/RC-dLH-II
Final no. particle images	129,052/116,858
Map resolution (Å, FSC = 0.143)	2.3/2.3
Symmetry imposed	C1
Specimen temperature	~80 K
Particle box size	(600 px)^2^ at 0.732 Å/px
Model refinement and validation	
Refinement package	COOT, PHENIX, ISOLDE, AlphaFold 3
Initial model	PDB 7O0W + AlphaFold 3 prediction
Model composition	
Non-hydrogen atoms	49,751/49,759
Protein residues	5,043/5,049
Molecular weight (kDa)	648.69/648.81
Protein B factor (Å^2^)	60.43/61.24
Ligand B factor (Å^2^)	56.64/54.59
RMS deviations	
Bond length (Å)	0.004/0.003
Bond angle (°)	0.810/0.719
Validation	
MolProbity	1.58/1.15
Clashscore	7.70/7.72
Poor rotamers (%)	1.6/1.34
Cβ outliers (>0.25 Å deviation)	0.00/0.00
CaBLAM outliers (%)	0.69/1.0
Ramachandran plot	
Favored (%)	98.36/98.55
Allowed (%)	1.64/1.45
Disallowed (%)	0.00/0.00
Ramachandran *Z*-score	1.47/3.32 (whole)
PDB ID	9H19/9H22
EMDB ID	EMD-51760/EMD-51788

### Cryo-EM data processing

All image processing steps were performed in CryoSPARC version 4.2.1. Raw micrograph movies were corrected for beam-induced motion using patch motion correction. CTF parameters of motion-corrected micrographs were estimated using patch CTF. Particles were picked using blob picker (160−240 Å), NCC of 0.25+. Particles were downsampled by a factor of 2 and extracted from micrographs. Down-sampled particles were used in two rounds of 2D classification (number of classes = 100, batch size per class = 1,000, number of online-EM iterations = 100, and number of final full iterations = 5). Particles from featureless, noisy, or poorly resolved classes were discarded. 2D classes revealed a mixture of monomeric and dimeric RC-dLH. Only particles from well-resolved 2D monomer classes were subjected to *ab initio* reconstruction (number of particles 282,874). Particles from the best-resolved *ab initio* class were subjected to homogeneous refinement to reach a consensus map. The map is locally refined using a local mask covering the RC-LH1 region only. Particles were then re-extracted without downsampling and used in non-uniform refinement followed by local refinement. 3D classification was carried out using the consensus map, resulting in two classes; each class was subjected to non-uniform refinement. The resulting particles and maps were subjected to global CTF refinement and reference-based motion correction. The motion-corrected particles were refined to give the final maps at 2.3 Å resolution for each of the two classes, with EMDB access IDs EMD-51760 and EMD-51788, respectively.

### Modeling and refinement

The overall shapes of the two contour maps looked similar: the RC is encircled by LH1 to form a RC-LH1, and the LHh surrounds this complex to form RC-dLH. The difference between these two maps is a relative 7.5° rotation of the LHh ring against the LH1 ring. In this case, the first class of particles (Model-I) was used for initial modeling. One of the RC-dLH complexes from *Gem. phototrophica* (PDB 7O0W) was rigid-body docked into the Model-I map as an initial model using ChimeraX. Amino acid sequences of all polypeptides were fitted into maps by AlphaFold 3 (21) and ModelAngelo (39). This fitting confirmed that the RC H-subunit is split into two parts: a transmembrane subunit Ht and a cytoplasmic side subunit Hc. The following ligands— bacteriochlorophyll, bacteriopheophytin, gemmatoxanthin, dodecyl-maltoside, phosphoethanolamine, heme, water, cardiolipin, menaquinone, and spirilloxanthin—were modeled using COOT (40). The contour map of the RC-dLH from *Gem. groenlandica* clearly shows that there are two BChl *a* molecules and one carotenoid molecule in both LH ring subunits. However, the flexibility of the BChl *a* tail in the complex hampers distinguishing its type, geranylgeranyl or phytol. A BChl *a* molecule with a phytol tail was modeled supported by HPLC analysis, showing that this tail of BChl *a* is the dominant component. For the same reason, the carotenoid gemmatoxanthin was used for all carotenoid modeling except for spirilloxanthin in the RC due to an obvious better fit to the density. Other ligands, including lipids, detergents, water, etc., were inserted into the map based on fit to the cryo-EM map. After real-space refinement was performed in COOT, protein stereochemistry was initially refined in ISOLDE (41). By rotating LHh 7.5°, Model-II was created from Model-I. Both models were submitted to Phenix (42) for final refinement and validation. Atomic model refinement statistics are listed in Table 1. Final models were deposited in the Protein Data Bank with deposition IDs PDB 9H19 for Model-I and 9H22 for Model-II. A data processing workflow chart is given in Fig. S2, and the difference between the two models is illustrated in Fig. 4.

### Alignments

Whole genomes of phototrophic strains of *Rvi. gelatinosus* IL 144, *Pgt. spongiicola*, *Gem. phototrophica,* and *Gem. groenlandica* were downloaded from NCBI (July 2024). Genes forming the PGC were analyzed in Geneious Prime (version 2024.0.5). Polypeptide sequences of PufB, PufA, PufA1, PufA2, and PufM were extracted from whole genomes of these species as appropriate. For each of the polypeptides, multiple sequence and pairwise alignments were done in Geneious Prime (version 2025.0.3) using MAFFT alignment (43), and the percentage of protein sequence identities was computed for multiple sequence alignments as well as pairwise alignments. Figure 8a showing the PGC alignments and Fig. 8b and Fig. S6 showing polypeptide alignments were made in Inkscape 0.92.4.

## Data Availability

Atomic coordinates and the cryo-EM density maps have been deposited in the PDB under accession numbers 9H19 and 9H22 and in the EMDB under accession numbers EMD-51760 and EMD-51788. All data needed to evaluate the conclusions in the paper are present in the paper and/or the supplemental material.

## SUPPLEMENTAL MATERIAL

Supplemental Figures msystems.01094-25-s0001.pdf Figures S1 to S6.

ASM does not own the copyrights to Supplemental Material that may be linked to, or accessed through, an article. The authors have granted ASM a non-exclusive, world-wide license to publish the Supplemental Material files. Please contact the corresponding author directly for reuse.
