# Supplementary material for: Two solutions for efficient light-harvesting in phototrophic Gemmatimonadota
Source: mSystems. 2025 Dec 4;11(1):e01094-25. doi: 10.1128/msystems.01094-25 (PMC12817894; doi:10.1128/msystems.01094-25)
Supplement: Supplemental Figures — Figures S1 to S6. [file msystems.01094-25-s0001.pdf]

Supplementary Information

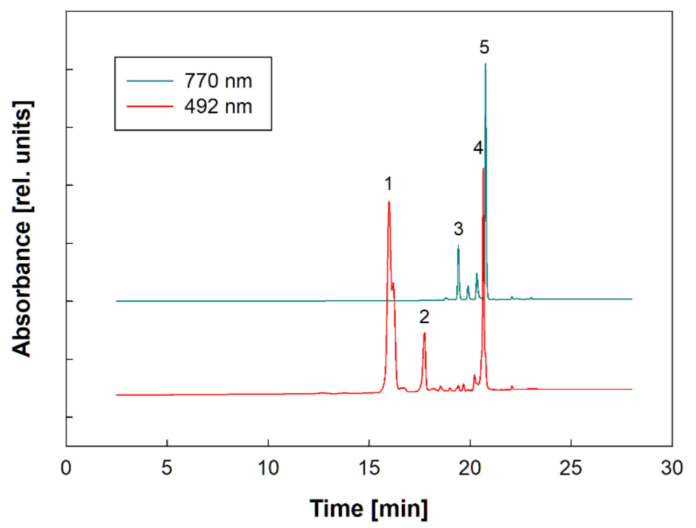

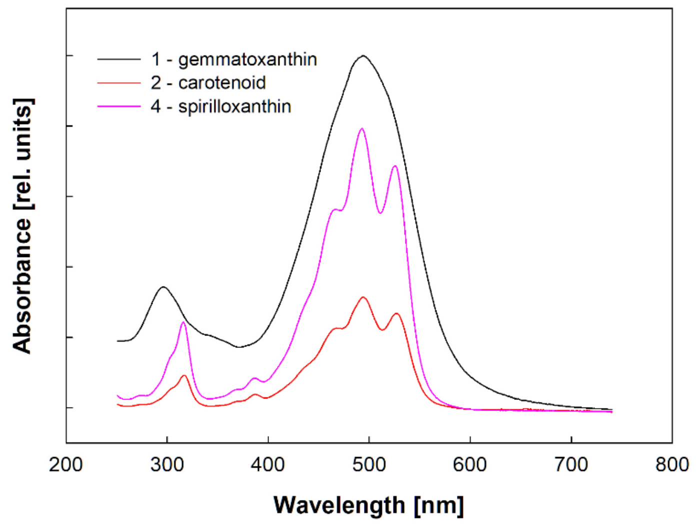

Figure S1.

HPLC elution profile of pigments extracted from the *Gem. groenlandica* RC-dLH complex. The red trace was detected at 492 nm for carotenoids and the cyan trace at 770 nm detects BChl. Identified peaks; 1 - gemmatoxanthin (two isomers), 2 - unknown spirilloxanthin-like carotenoid, 3 - bacteriochlorophyll *a* with geranylgeranyl sidechain, 4 – spirilloxanthin, 5 -bacteriochlorophyll a with phytol sidechain. The respective spectra for the carotenoid peaks are given below.

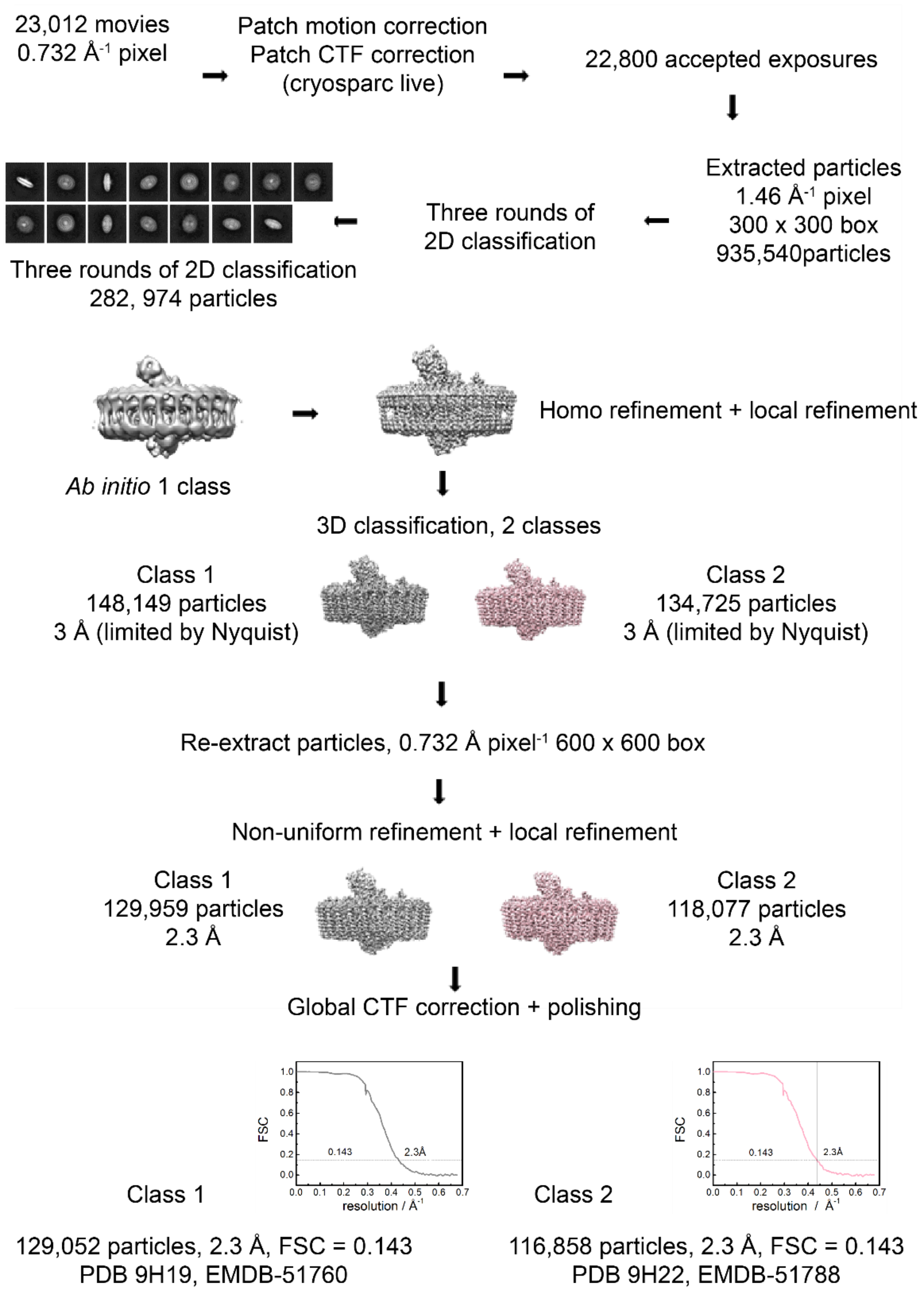

Figure S2.

Data processing workflow. The data processing workflow used to obtain the two *Gem. groenlandica* RC-dLH particles. The difference between Model-I and Model-II is shown in Figure 5

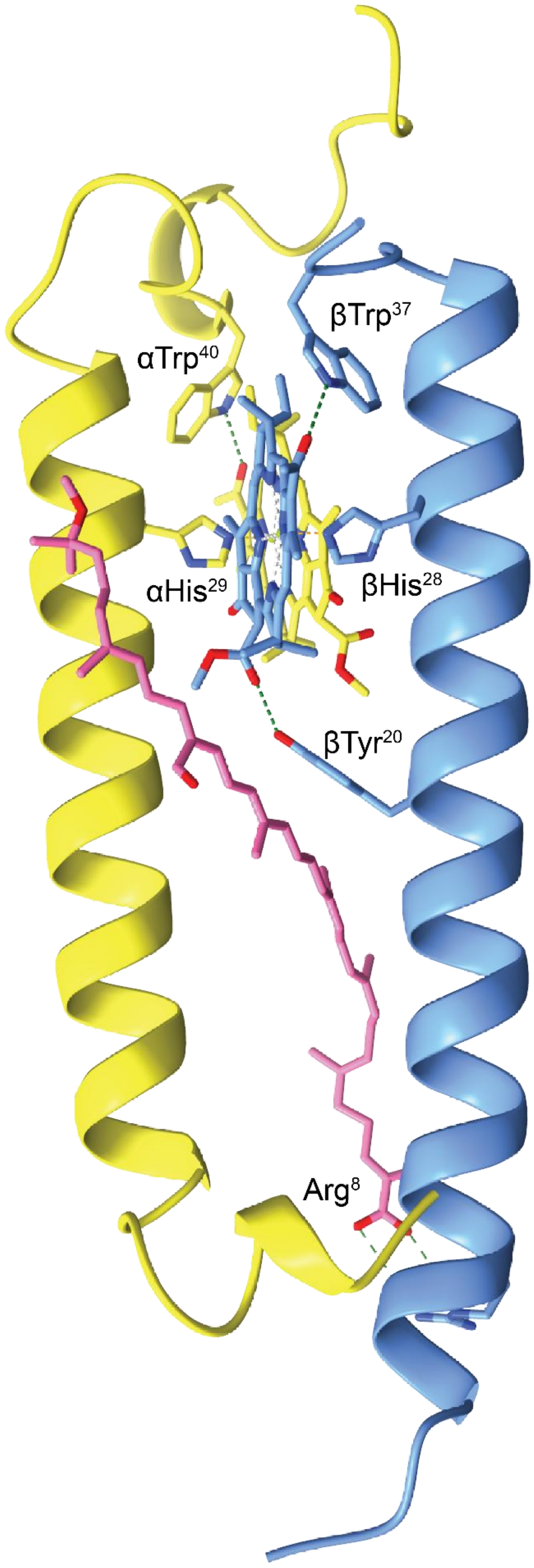

Figure S3.

An αβ-heterodimer from the *Gem. groenlandica* LH1 ring with the periplasmic side at the top and cytoplasmic side at the bottom. The β-polypeptide is colored cornflower blue and the α- polypeptide in yellow. The BChl pigments are colored to match the binding polypeptide with the carotenoid gemmatoxanthin in pink. Three H-bonds (from α-Trp^{40} to the α-BChl C3^{1} keto group, from β-Trp^{37} to β-BChl C3^{1} keto group and from β-Tyr^{20} to the β-BChl C13^{3} ester group) stabilises the BChl. These interactions are essentially identical between the *Gem. groenlandica* LH1 ring and the *Gem. phototrophica* LH1 ring, with the possible exception of the H-bonds between Arg^{8} and the end of the gemmatoxanthin molecule. The BChl phytol tails have been omitted for clarity.

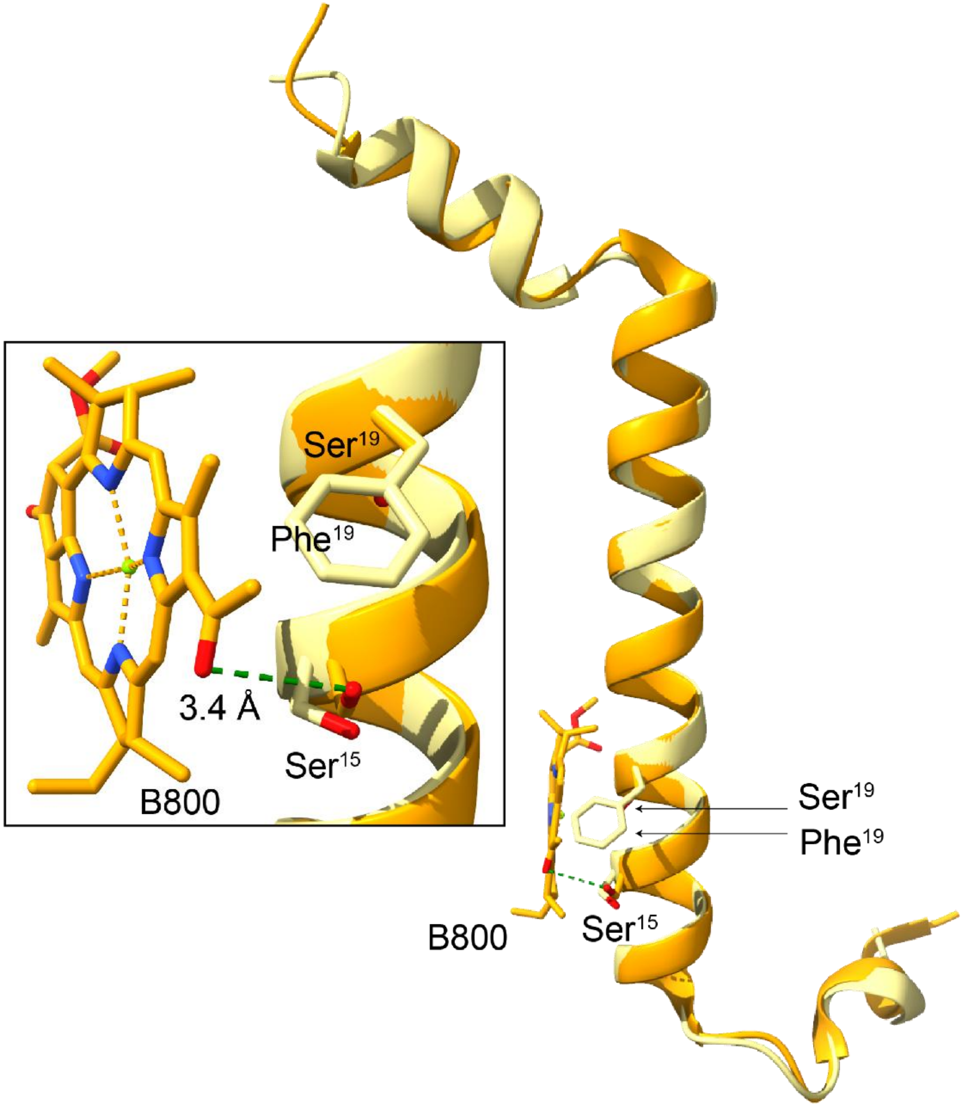

Figure S4.

An α-subunit from LHh of *Gem. phototrophica* (orange) and *Gem. groenlandica* (Model I, khaki) superimposed using the ChimeraX Matchmaker function. This superimposition was used to make the panel in Figure 6c. The two subunits overlie each other extremely well, especially in the hydrophobic, trans-membrane region. The insert shows a close-up of the B800 region to better illustrate the different orientations of Ser^{19} and that the bulky sidechain Phe^{19} is not responsible for the lack of B800 in *Gem. groenlandica*. The periplasmic side is at the top and the cytoplasmic side is at the bottom.

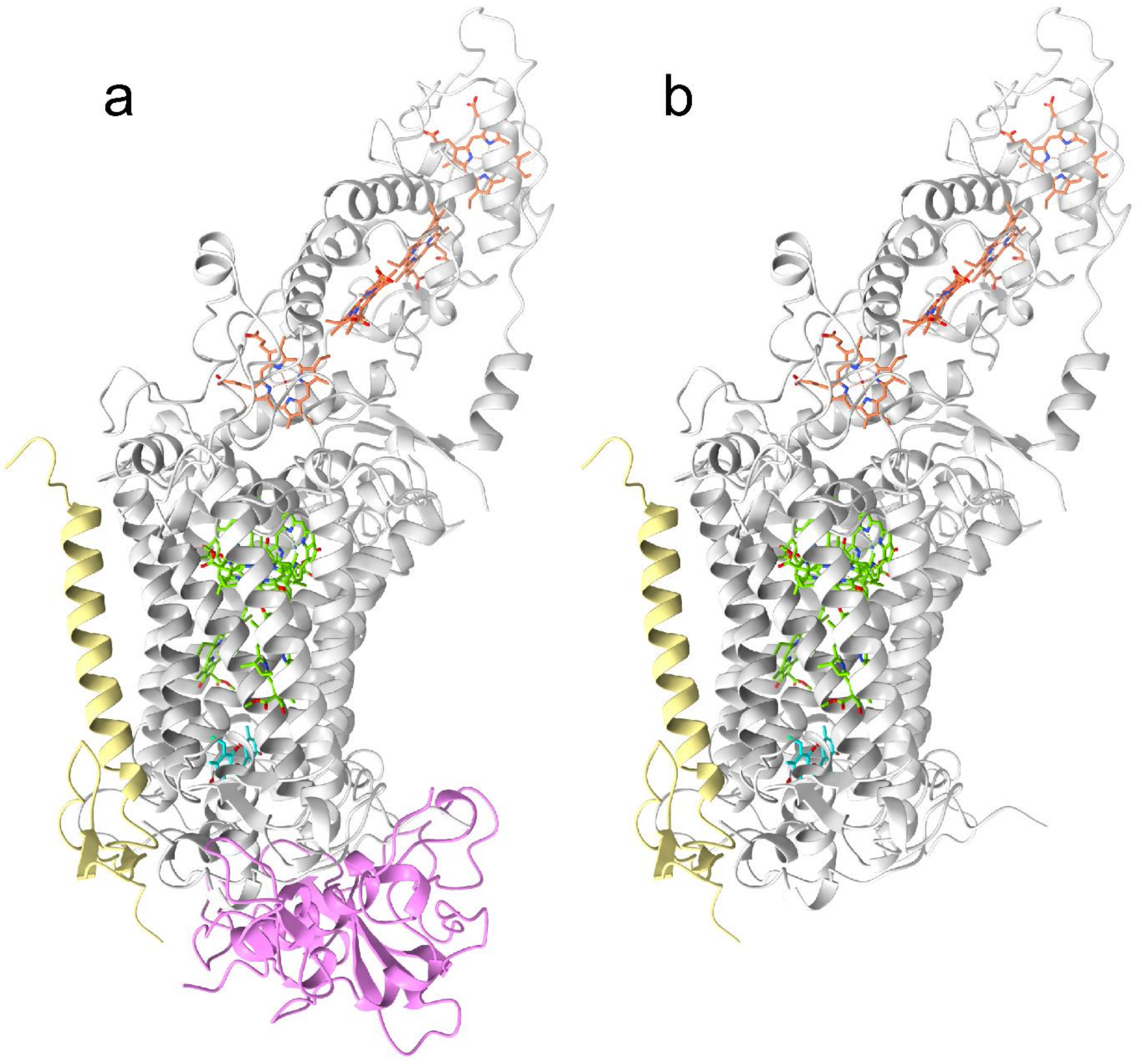

Figure S5.

The split H subunit in the RC. (a) H_t (wheat) and H_c (warm pink) subunits of the *Gem. groenlandica* RC-dLH complex. The L, M and C (bound Cytochrome) subunits are shown in grey. The haem groups within C are shown in salmon, the BChls, bacteriopheophytin in lime green and quinones in cyan (b) The same structural motif is used to figuratively illustrate the loss of the Hc subunit. The BChl tails have been removed for clarity.

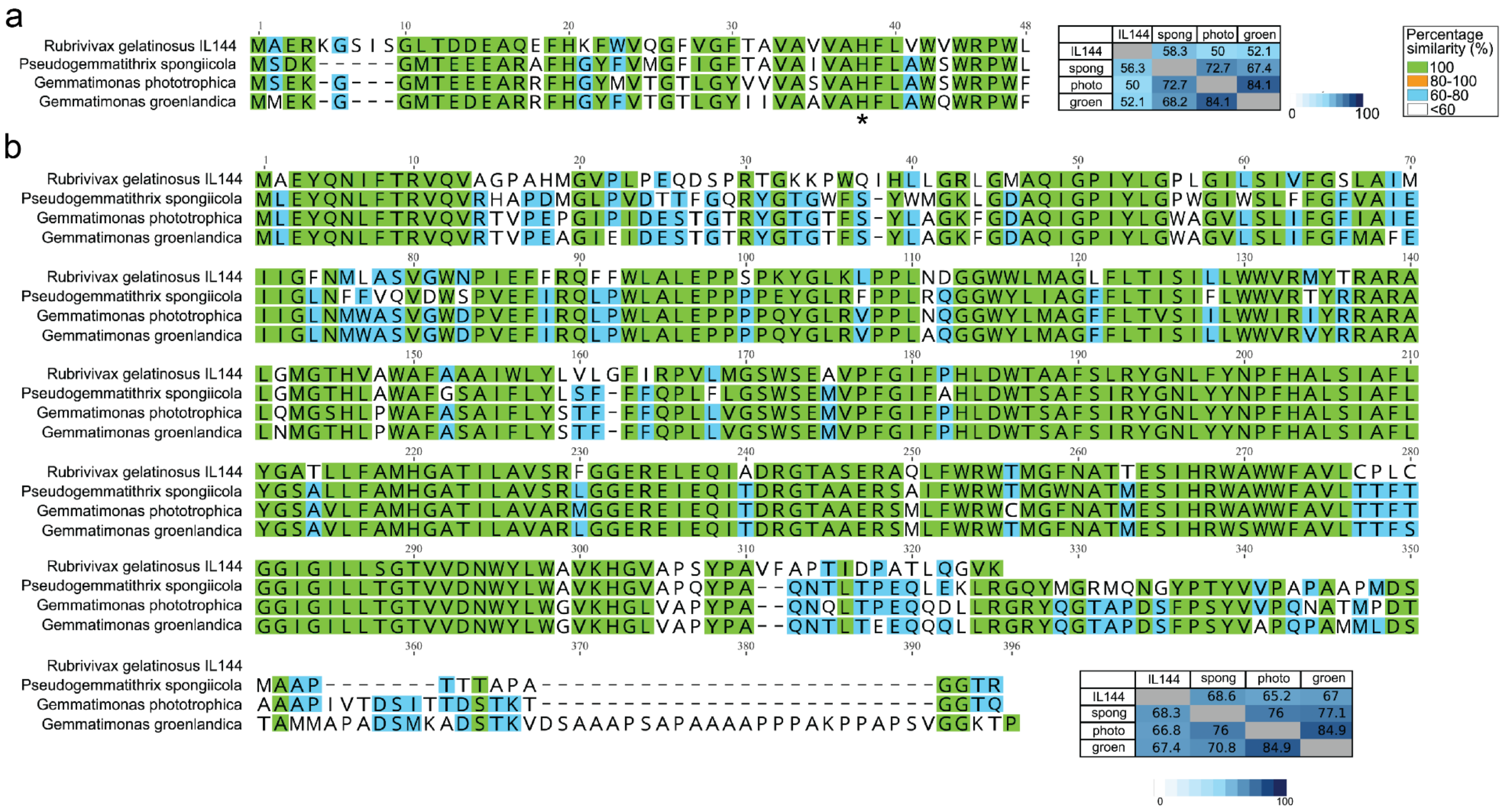

a
Rubrivivax gelatinosus IL144
Pseudogemmatithrix spongiicola
Gemmatimonas phototrophica
Gemmatimonas groenlandica
IL144 spong photo groen
IL144 58.3 50 52.1
spong 56.3 72.7 67.4
photo 50 72.7 84.1
groen 52.1 68.2 84.1
0 100
Percentage similarity (%)
100
80-100
60-80
<60
b
Rubrivivax gelatinosus IL144
Pseudogemmatithrix spongiicola
Gemmatimonas phototrophica
Gemmatimonas groenlandica
IL144 spong photo groen
IL144 68.6 65.2 67
spong 68.3 76 77.1
photo 66.8 76 84.9
groen 67.4 70.8 84.9
0 100

Figure S6.

(a) Alignment of PufB polypeptide sequences of *Rvi. gelatinosus* IL144 with *Pgt. spongiicola*, *Gem. phototrophica* and *Gem. groenlandica* (b) PufM polypeptide sequence alignment of *Rvi. gelatinosus* IL144, *Pgt. spongiicola*, *Gem. phototrophica* and *Gem. groenlandica*. The table for each alignment shows the percentage identities for each sequence. The left side is percentage identities computed by multiple sequence alignment and the right side shows percentage of pairwise identities. A color coded legend is shown beside each table with white representing the lowest identity and dark blue the highest. A color coded key for the residue percentage similarity is also given. In both *Gem. phototrophica* and *Gem*. *groenlandica*, βHis^{34} interacts with the central Mg^{2+} ion of β-BChl so this residue is marked with an asterisk.
